# Colorless Tincture of Iodine

**Published:** 1868-06

**Authors:** Hubert Primm

**Affiliations:** Professor of Practical Pharmacy in the St. Louis College of Pharmacy.


### ARTICLE IX.

### *Colorless Tincture of Iodine.*

By HUBERT PRIMM, Ph. D., Professor of Practical Pharmacy in the St. Louis College of Pharmacy.

Having had frequent calls for a colorless tincture of iodine, and the formula in use presenting decided therapeutic objections, I have devised the one which will be found at the close of this article. There are two formulæ in general use, the first being made as follows:

| | |
|---|---|
| Iodine - - - - - - - | ℥ j. |
| Alcohol - - - - - - | f ℥ xij. |
| Water of ammonia - - - - - | f ℥ iv. |

Dissolve the iodine in the alcohol, and add the water of ammonia. Let stand until bleached and filter. The result of this process is the formation of an alcoholic solution of iodide of ammonia, with perhaps some iodate, and contaminated with the presence of caustic ammonia, which prevents the colorization of the product.

It is obvious that the use of this preparation can but be limited, for if we recollect that properly prepared aq. ammonia contains ten per cent. of ammonia, it requires but little forethought to see that there would arise many cases in which though the use of tr. of iodine would be indicated, yet owing to the very caustic nature of this preparation, its employment would be dangerous. The second formula is that of Dr. Percy Boulton, and is made as follows:

R.—Compound tincture of iodine - - 46.5 grs
Liquid carbolic acid - - - - 6 drops.
Glycerine - - - - ℥ vij—45.5 grs.
Distilled water - - ℥ iv—ʒ v—37.5 "
*M.*

This formula presents some very valuable therapeutic advantages, combining, as it does, deobstruent qualities of the iodine with the peculiar antiseptic properties of the carbolic acid, and, no doubt, in practice will fill up a vacuum that has long existed, as there are many instances in which the combination of iodine and carbolic acid would be peculliarly advantageous.

The objection that can be urged against its use lies in the fact that, to many, especially to women, the intolerable odor of the discoloring agent is as disgusting as is the disfiguring effects of the officinal tr. of iodine, besides which I have not as yet, met with any sample of carbolate of iodine which is perfectly colorless—they were all more or less of a light yellow hue. It will also be perceived that there is but a small percentage of iodine in each drachm of the preparation, which rather unfits it for external use.

In the formula herewith submitted, the writer, without, arrogating to himself much claim to originality, thinks that by the use of his process many of the objections that are urged against the use of the formulæ above presented will be obviated. It is as follows:

*R.*—Tincture of iodine, U. S. P. - - f ʒ vj.

Distilled water - - - - - f ʒ ii.

Hyposulphite of soda - - - 108 grs.

Triturate the soda with the water, and add the tr. of iodine gradually, with constant stirring; when the process is completed, filter.

The resulting tincture is a clear and limpid solution, possessing no causticity beyond that naturally pertaining to all alcoholic solutions of iodine; and as far as as I have been able to learn from those who have used it, has all resolvent, absorbent and deobstruent properties that belong to the officinal tincture of iodine.

In conclusion, the writer of this is well aware that the preparation of a colorless tinct. of iodine, which would represent iodine *per se*, without the formation of a new chemical compound is, as yet, a chemical impossibility; but he does claim that as long as the medical profession have to have recourse to combinations of iodine with discoloring agents, the one which will present it the freest from causticity and odor is the best.—*St. Louis Medical Reporter.*

